# Lipid Nanoparticles-Encapsulated YF4: A Potential Therapeutic Oral Peptide Delivery System for Hypertension Treatment

**DOI:** 10.3389/fphar.2019.00102

**Published:** 2019-02-19

**Authors:** Shengnan Zhao, Jinhua Li, Yang Zhou, Lingjing Huang, Yanfei Li, Juanjuan Xu, Chunmei Fu, Xia Guo, Jian Yang

**Affiliations:** ^1^School of Applied Chemistry and Biological Technology, Shenzhen Polytechnic, Shenzhen, China; ^2^Key Laboratory of Birth Defect and Related Disorders of Women and Children, Department of Pediatric Hematology/Oncology, West China Second University Hospital, Sichuan University, Chengdu, China; ^3^Key Laboratory of Drug Targeting and Drug Delivery System (Ministry of Education), West China School of Pharmacy, Sichuan University, Chengdu, China

**Keywords:** lipid nanoparticles, antihypertensive peptide, hypertension, oral administration, sustained release, continuously antihypertensive effect

## Abstract

Drugs are administered orally in the clinical treatment of hypertension. Antihypertensive peptides have excellent angiotensin converting enzyme inhibitors activity *in vitro*. However, the poor oral bioavailability and therapeutic effect of antihypertensive peptides were mainly caused by rapid degradation in gastrointestinal and the short circulation time in blood, which remain to be further optimized. Therefore, the novel oral peptide delivery system is urged to improve the oral absorption and efficacy of peptide drugs. In this work, Tyr-Gly-Leu-Phe (YF4)-loaded lipid nanoparticles (YF4-LNPs) combined the advantages of polymer nanoparticles and liposomes were developed, which could greatly enhance the oral bioavailability and ameliorate the sustained release of peptide drug. YF4 loaded nanoparticles (YF4-NPs) were firstly prepared by a double-emulsion internal phase/organic phase/external phase (W1/O/W2) solvent evaporation method. YF4-NPs were further coated by membrane hydration-ultrasonic dispersion method to obtain the YF4-LNPs. The optimal YF4-LNPs showed a small particle size of 227.3 ± 3.8 nm, zeta potential of -7.27 ± 0.85 mV and high entrapment efficiency of 90.28 ± 1.23%. Transmission electronic microscopy analysis showed that the core-shell lipid nanoparticles were spherical shapes with an apparent lipid bilayer on the surface. Differential scanning calorimetry further proved that YF4 was successfully entrapped into YF4-LNPs. The optimal preparation of YF4-LNPs exhibited sustained release of YF4 *in vitro* and a 5 days long-term antihypertensive effect *in vivo*. In summary, the lipid nanoparticles for oral antihypertensive peptide delivery were successfully constructed, which might have a promising future for hypertension treatment.

## Introduction

Nanotechnology has been widely used to improve the oral absorption and therapeutic efficacy of small molecule peptide drugs, which have shown tremendous potentials but great challenges (Olbrich et al., 2001; Tan et al., 2009; Li et al., 2013; Yang et al., 2013; Thi et al., 2015; Wang et al., 2018). Poly-(lactic-co-glycolic) acid (PLGA) nanoparticles have attracted much attention, owing to the unique properties of biodegradability, biocompatibility, and sustained release (Musumeci et al., 2006). Liposomes also have been applied for drug delivery due to the superior biocompatibility, drug absorption and nontoxicity (Wang and Liu, 2013). Unfortunately, both of them have some unsatisfied disadvantages in peptide delivery, such as leakage and safety issues, which seriously limited the delivery efficiency. It is necessary to deal with these problems for improving the oral delivery efficiency of peptides (Luo et al., 2006).

Lipid coating PLGA nanoparticles system (lipid nanoparticles, LNPs) has not only combined the advantages of polymer nanoparticles and liposomes but efficiently avoided the defects of them (Muller and Keck, 2004; Alavi et al., 2017). The dual advantages of the particles and vesicle make it an excellent oral drug carrier with high biocompatibility and sustained release (Xie et al., 2018). In this system, drugs can be efficiently encapsulated in the nanoparticles core and/or the lipid bilayers, resulted in increased drug load capability. The drug diffusion rate can be delayed by the polymer core. Additionally, the stability of the LNPs can be further increased by the lipid shell (Nogués et al., 2006). However, it is still challenging to achieve high encapsulation efficiency (EE) and decent particle size when incorporating hydrophilic drugs into LNPs.

Hypertension has become a strong risk factor for cardiovascular disease and affected almost 2 billion people. In detail, it has highly correlated with the heart attack, cerebral hemorrhage, stroke, kidney failure and blindness (Kjeldsen, 2017). Angiotensin-converting enzyme (ACE), located in various tissues, is potent to affect the cascade of process that trigger the increasing of blood pressure. Hence, ACE inhibitors (ACEIs) are commonly used to decrease blood pressure (Girgih et al., 2016). However, the synthetic ACEIs were reported to produce negative side effects such as dry cough, angioedema, itch, diarrhea (Tenenbaum et al., 2000; Abassi et al., 2009), and renal impairment. Alternatively, natural antihypertensive peptide fragments have gradually entered the field of human vision due to the sound security.

YF4, a polypeptide extracted from milk protein, has been reported for the opioid activity for the first time in 1986 (Yoshikawa et al., 1986). Other research groups have conducted other pharmacological studies and found that it had hypotensive activity as well (Espejo-Carpio et al., 2013). However, the antihypertensive peptides are usually accompanied by extremely poor stability and easily degraded by gastric acid and pepsin in the gastrointestinal (GI). At present, the existing peptide drugs in clinical are given mainly by injection (Boelsma and Kloek, 2010; Majumder et al., 2013). Because of the short circulation time (Agrawal et al., 2015) and degradation of polypeptide drugs in the GI, the patients need to be injected frequently which results in the poor compliance (Cleland et al., 2012). To address these issues, novel oral peptide delivery systems are urged to improve their stability in the stomach and intestines in order to improve its therapy effect with a convenient and economic way (Niu et al., 2018).

In the present study, we chose the antihypertensive peptide YF4 as a model drug to systematically optimize the lipid nanoparticles and investigate it’s potential in oral peptide delivery. The formulation parameters of YF4-LNPs were systematically investigated. The physic-chemical properties including particle size, surface charge, EE, Transmission electron microscope (TEM), Differential scanning calorimeter (DSC), and *in vitro* release profile were then characterized systemically. Additionally, the *in vivo* antihypertensive efficacy was assessed on spontaneously hypertensive rats.

## Materials and Methods

### Materials

YF4 (purity > 99%) was gained from Phtdpeptides Co., Ltd. (Zhengzhou, China). PLGA (MW = 15 kDa; LA/GA = 75:25) was purchased from Jinan Daigang Biomaterial Co., Ltd. (Jinan, China). Poly (vinyl alcohol) (PVA, MW = 30–70 kDa, HD, 80%) and TPGS were obtained from Sigma-Aldrich (St. Louis, MO, United States). Soybean phosphatidylcholine (SP) was gained from Shanghai A.V.T. Pharmaceutical Co., Ltd (Shanghai, China). Carbomer 996 was supplied by Hang Zhou Carbokar Import & Export Co., Ltd. (Hang Zhou, China). Poloxamer 188 was acquired from BASFSE (Germany). mPEG_2000_-Chol (purity > 98.6%) was synthesized by State Key Laboratory of Biotherapy, Sichuan University. Cholesterol was purchased from Shanghai source poly Biotechnology Co., Ltd.). All other reagents were of analytical grade and were used unless otherwise stated.

### Animals

Twelve weeks old male spontaneously hypertensive rats (SHRs) with a circa weight between 250 and 320 g were purchased from Beijing Vital River Laboratory Animal Technology Co., Ltd. After a week of adaption, animals were admitted to experiments (six per group). Animals were maintained under 12 h dark and light cycles at controlled temperature, 55% humidity, 22°C. The rats were free access to food and water. All the experiments were approved and supervised by the State Key Laboratory of Biotherapy Animal Care and Use Committee (Sichuan University, Chengdu, Sichuan, China). In this study, groups of rats were used for the oral administration of saline group, Captopril group vs. YF4 group, YF4 vs. YF4-NPs vs YF4-LNPs.

### Preparation of YF4-LNPs

YF4-NPs were prepared by an easy and controllable method. The YF4-NPs were further coated by membrane hydration-ultrasonic dispersion method to obtain the lipid nanoparticles (YF4-LNPs). Firstly, the core of YF4-LNPs (YF4-NPs) was prepared by a double-emulsion internal phase/organic phase/external phase (W1/O/W2) solvent evaporation method (Yu et al., 2016). In brief, 50 μL YF4 (10 mg/mL) deionized water solution was used to form the inner aqueous phase. The 1 mL organic phase (mixed solution of acetone and acetonitrile, initial volume ratio was 5:1) containing PLGA was used as the organic phase, and then mixed with the inner aqueous phase by probe sonication in ice bath to form the primary W1/O emulsion. The primary emulsion was then added to the 4 mL external phase solution and further sonicated to obtain the final W1/O/W2 double emulsion. The organic phase in the ultimate emulsion was rapidly removed by evaporation under vacuum at 37°C to get YF4-NPs. At the same time, SP (lipid), cholesterol (Chol) and mPEG_2000_-cholmixture (the mass ratio was 4:1:0.25) were dissolved in 2 mL chloroform at a certain proportion. The organic solvents were subsequently removed using a rotary evaporator (R-201Shanghai Shen Shun Biological Technology Co., Ltd.) to produce a thin film of lipid at 37°C. The lipid film was hydrated with 4 mL prepared YF4-NPs for 1 h at 60°C to obtain a suspension. YF4-LNPs were finally acquired by Ultrasonic cell crushing and isolating machine (VCX130, American Systems on ICs).

### Characterization of YF4-LNPs

#### Particle Size and Zeta Potential

The mean particle size, size distribution and ζ potential were measured by Zetasizer (Zetasizer Nano-ZS 90; Malvern Instruments Ltd., Malvern, United Kingdom) at 25°C. The prepared YF4-LNPs were diluted with deionized water and experiments were conducted in triplicate. All the data were presented as mean ± SD.

#### Entrapment Efficiency and Drug Loading

Briefly, the obtained supernatant by ultrafiltration centrifuging the colloidal suspension during the preparation of YF4-LNPs was stored to determine the EE and drug loading capacity (DL) of YF4-LNPs as previously described (Yu et al., 2016). The untrapped YF4 in supernatant was determined by high performance liquid chromatography (HPLC, Waters Alliance 2695) to calculate the amount of YF4 unpackaged into YF4-LNPs. Analyses were performed in triplicate and the values were expressed as mean ± SD. EE and DL of YF4 were calculated as following formulas:

$$\begin{array}{c} \mathrm{EE}\%\;=\left(\frac{TotalYF4amount-AmountofYF4insupernatant}{TotalYF4amount}\right)\;\times \;100\\ \mathrm{DL}\%\;=\left(\frac{TotalYF4amount-AmountofYF4insupernatant}{\mathrm{Totalweightoflipidnanoparticles}}\right)\;\times \;100 \end{array}$$

#### Stability

YF4-LNPs were stored at 4°C for two weeks to investigate the preliminary stability. The changes of particle size and EE of YF4-LNPs were examined by the methods described above. Analyses were performed in triplicate and the values were expressed as mean ± S.D.

#### Appearance and Morphology

The appearance and Tyndall effect of YF4-LNPs were observed by a digital camera. The morphology of the YF4-LNPs was examined by TEM (H-600, Hitachi, Japan). In brief, the prepared samples were diluted to proper concentration by deionized water and then were placed on a copper electron microscopy grid and negatively stained with 2wt% phosphotungstic acid solution for 30 s for observation.

#### Differential Scanning Calorimetry (DSC)

The physical state of YF4 loaded in YF4-LNPs was verified by DSC (200PC, Netzsch, Karlsruhe, Germany). Freeze-dried YF4-LNPs, blank LNPs, YF4, and the physical mixture of blank LNPs and free YF4 with the same mass ratio as those in YF4-LNPs were heated from 75 to 250°C at a heating ramp of 20°C/min under nitrogen atmosphere at a flow rate of 50 mL/min.

#### *In vitro* Release Studies

The *in vitro* release profiles of YF4-LNPs were investigated in PBS buffer at pH 1.0 (simulated gastric fluid), pH 4.5, pH 6.8 (simulated different intestinal fluids), and pH 7.4 (physiological pH condition) by dynamic dialysis method (Boelsma and Kloek, 2010). In brief, free YF4 solution, YF4-NPs, and YF4-LNPs were first dispersed in release media in dialysis bags (MWCO 3000) and then were shaken at 37°C with a speed of 100 rpm. One milliliter buffer was removed and replaced with equal volume fresh release medium at 0, 2, 4, 8, 12 h, respectively. The content of YF4 was measured by HPLC after centrifugation for 10 min at 13,000 rpm as described in section of “Entrapment efficiency and drug loading.” Analyses were performed in triplicate and the values were expressed as mean ± S.D.

#### *In vivo* Antihypertensive Efficacy

Before the experiment, we first verified the antihypertensive activity of YF4 in SHRs. Simultaneously, the captopril and the saline water were used as the positive control and control group, respectively. SHRs were randomly divided into three groups (*n* = 6). Control group were treated with 0.9% saline water, and the other groups were treated with captopril (5 mg/kg) or the same dose of YF4 dissolved in saline water. All rats were administered at a single oral dose. Blood pressure was indirectly recorded using the tail-cuff method (BP-2010A, Softron Beijing Biotechnology, China) at each of the following times: 0, 2, 4, 8, 12, and 24 h after administration.

In YF4-LNPs pharmacodynamics study, the SHRs were randomly divided into three groups (*n* = 6). Control group were treated with 0.9% saline water, the other groups of SHRs were treated withYF4 and YF4-LNPs. All the groups of rats were given the same dose at 0.8 mg/kg body weight. Blood pressure was indirectly recorded as described above. Aside from free YF4 group, all the other groups were needed to detect blood pressure at more time points every day after administration until the pressure reached to normal level. All the SBP were measured for three times to get the average value at each time point.

#### Statistical Analysis

The obtained data were analyzed using the Graph Pad Prism 5. Data were analyzed by one-way analysis of variance. *p* < 0.05 was considered a statistically difference, and *p* < 0.01 was considered a statistically significant difference.

## Results

### Characterization of YF4-LNPs

**FIGURE 1:**
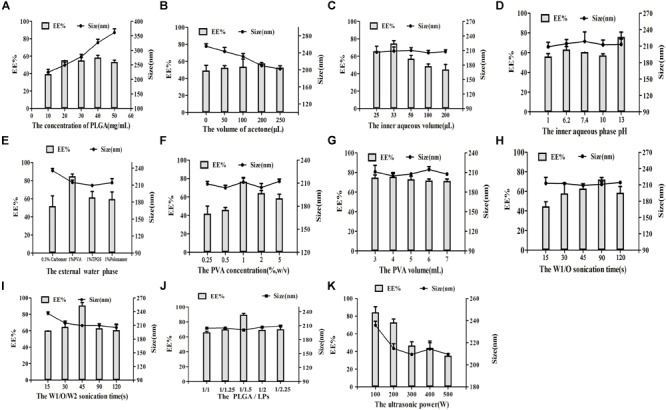
Effect of various processing parameters on the particle size (nm) and EE of YF4-LNPs. **(A)** The concentration of PLGA, **(B)** The volume of acetone, **(C)** The volume of the inner aqueous medium, **(D)** The pH, **(E)** The external water phase, **(F)** The concentration and **(G)** The volume of PVA, **(H)** The ultrasonic time of W1/O and **(I)**W1/O/W2, **(J)** The ratio of PLGA/LPs, and **(K)** The ultrasonic power.

**FIGURE 2:**
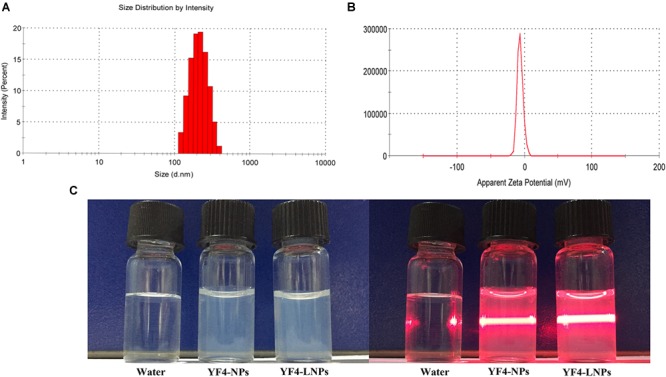
Physical-chemical properties of YF4-LNPs. **(A)** Size distribution. **(B)** Zeta potential. **(C)** The appearance of YF4-LNPs.

Several factors that could affect the features of YF4-LNPs were systematically optimized to produce the desirable YF4-LNPs, including the PLGA concentration, the volume of acetone in the organic phase, the volume of the inner aqueous medium, the pH of the aqueous medium, the external water phase, the PVA concentration, the PVA volume, the ultrasonic time, PLGA to lipid material ratio, and the ultrasound power. Firstly, the influence of PLGA concentration on the EE and particle size was investigated in Figure 1A. The EE and the particle size were positively correlated with the PLGA amount within the range of 10–50 mg. Figure 1B showed that the EE was dually increased and then suddenly decreased when acetone volume was above 200 μL while the particle size was negatively correlated with the increasing of acetone volume in the organic phase. In Figure 1C, the EE firstly increased and then decreased dramatically with the increase of inner aqueous volume, whereas the particle size was negligibly affected by the volume of internal phase. As seen in Figure 1D, the EE occurred a sudden enlargement when the pH reached 13, whereas there were no obvious changes in particle size. As shown in Figure 1E, the PVA was used as the external water phase, both the EE and the particle size were desirable. Figure 1F illustrated the influence of the PVA concentration on EE and particle size. As PVA concentration increased, the EE firstly increased dramatically and then decreased over 1%. Simultaneously, the particle size was observed to slightly fluctuate as PVA concentration changed. As the volume of PVA increased, a slight downward trend of EE and a fluctuation of particle size were observed in the Figure 1G. In Figure 1H, as internal phase/organic phase (W1/O) ultrasonic time was prolonged, the EE increased dually firstly and then dropped, while particle size was relatively stable. Furthermore, the effect of the ultrasonic time of internal phase/organic phase/external phase (W1/O/W2) on EE and particle size was also investigated in Figure 1I. EE was firstly increased under short-term ultrasound condition, but was suddenly compromised when the ultrasonic time was beyond 45 s, while the particle size decreased with the ultrasonic time prolonged. Different ratios of PLGA vs. lipid bilayer materials (PLGA/LPs) were investigated in Figure 1J. The EE was also significantly affected by the PLGA/LPs ratio, while the particle size was relatively stable. Figure 1J showed that the formulations exhibited a better size and higher EE when the PLGA/LPs ratio was 1:1.5. It was shown in Figure 1K that with the ultrasound power increased from 100 to 500 W, both the EE and particle size exhibited a rapidly decreasing tendency. Overall, the optimal YF4-LNPs were demonstrated to significantly improve the drug incorporation with an EE of 89.88 ± 1.23% (*n* = 3) and DL of 2.18 ± 0.25%. The average particle size was 227.3 ± 3.8 nm with a narrow size distribution (PDI = 0.09 ± 0.02) (Figure 2A). The zeta potential of YF4-LNPs was slightly negative, with the value of -7.27 ± 0.85 mV (Figure 2B). The colloidal suspension was observed as slightly blue opalescence with strong Tyndall effect (Figure 2C).

**FIGURE 3:**
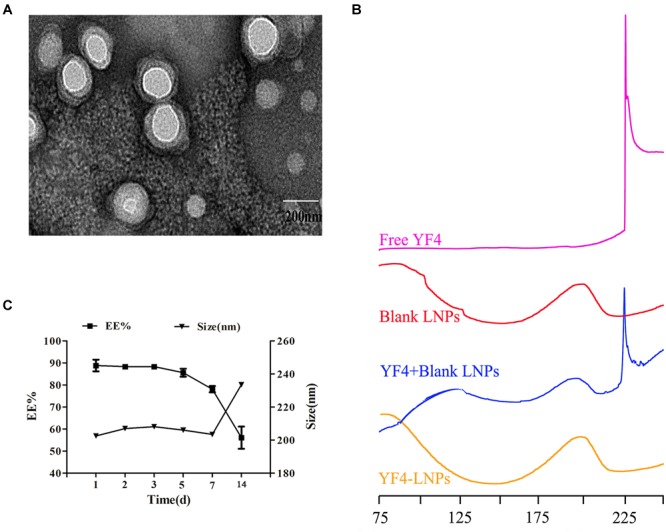
Characterization of optimal YF4-LNPs. **(A)** TEM image of YF4-LNPs. **(B)** DSC curves of Free YF4, Blank LNPs, Blank LNPs, and YF4 mixture and YF4-LNPs. **(C)** Change in EE and size of YF4-LNPs in 2 weeks stored at 4°C.

As shown in the TEM image in Figure 3A, YF4-LNPs were generally spherical, homogeneous and the lipid bilayer was well coated on the nanoparticles. In Figure 3B, the DSC analysis showed that the free YF4 displayed an obviously sharp endothermic peak at 225.6°C. The YF4-LNPs only presented two smooth endothermic peaks at 140.1°C, 205.2°C, with the absent sharp endothermic peak of free YF4. However, in the physical mixture group, there were three endothermic peaks at 145.3°C, 202.3°C, and the peak at 226.9°C, which represented the free YF4. All the obtained statistics suggested that YF4 was successfully encapsulated into the YF4-LNPs. The stability study displayed that YF4-LNPs remained stable without change of EE and size for at least 1 week (Figure 3C). When YF4-LNPs were stored for 2 weeks, the EE of YF4 got a sharp drop and the particle size became larger, which illustrated that YF4-LNPs could keep stable in 4°C for 1 week and were suitable for developing an oral administration.

### *In vitro* Release

**FIGURE 4:**
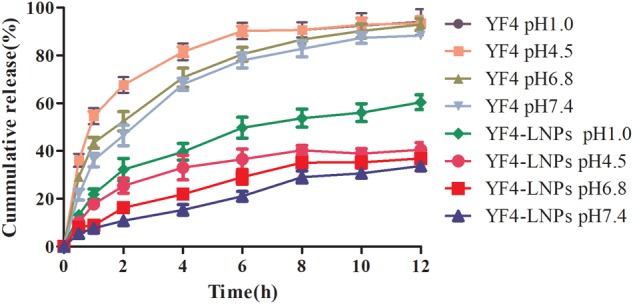
Release profiles of free YF4 and YF4-LNPs in different phosphate buffers (pH1.0, 4.5, 6.8, and 7.4).

*In vitro* release profiles of YF4 from YF4-LNPs were presented in Figure 4 and Supplementary Figure S2, the free YF4 exhibited a burst release, and over 80% of YF4 released in 6 h, while those loaded in YF4-LNPs were released gradually without apparent burst release in all of the release media. Within a 12 h period, compared with the approximately total release of free YF4 at pH 4.5, 6.8, only less than 40% of YF4 was released from YF4-LNPs. Besides, over more than 40% of YF4 was still packed in the YF4-LNPs while the free YF4 was quickly released in the acid-like solution in the stomach (pH 1.0). All the results illustrated that YF4 was efficiently packed in YF4-LNPs and might achieve a sustained release in small intestine.

### *In vivo* Antihypertensive Efficacy

**FIGURE 5:**
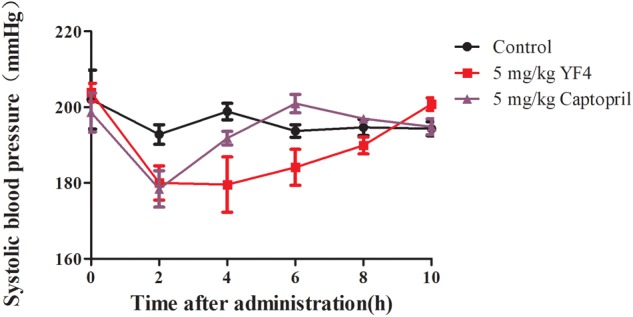
Antihypertensive effects of Free YF4 and Captopril in SHRs within 10 h by a single oral administration of 5 mg/kg.

To investigate the blood pressure lowing (BPL) effect of YF4, captopril was chosed as the positive control. As seen in the Figure 5, compared with saline group, the BPL effect of YF4 at a dose of 5 mg/kg presented at 2 h after a single oral administration was 23.0 mmHg, which was equal to captopril with the same dose. However, both of them showed the short BPL effect.

**FIGURE 6:**
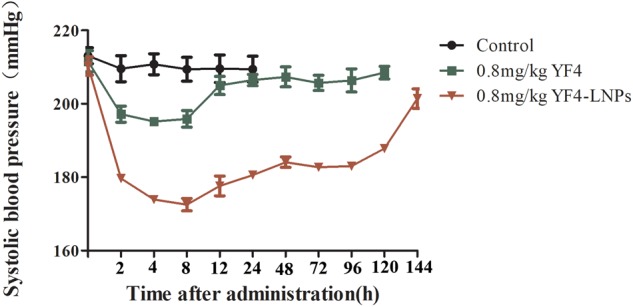
Antihypertensive effects of same doses of YF4and YF4-LNPs in SHRs within 144 h by a single oral administration.

Then we prepared the lipid nanoparticles encapsulated YF4 and further investigated the BPL effect of YF4-LNPs in SHRs. As Figure 6 showed, compared with saline group, YF4 and YF4-LNPs at a dose of 0.8 mg/kg all emerged a BPL effect. Administration of 0.8 mg/kg of YF4 signicantly decreased SBP by 15.6 mmHg at 4 h post-administration and SBP returned to the untreated level at 12 h post administration. And the decreased SBP between 2 and 120 h post administration was observed after treated by 0.8 mg/kg YF4 LNPs. YF4-LNPs at a dose of 0.8 mg/kg strikngly decreased the blood pressureby 43.5 mmHg about 2 h post administration. Amazingly, even after 120 h, obviously BPL effect was still seen as a significantly reduction of 20.5 mmHg. The SBP returned to the initial level at 144 h post administration.

## Discussion

The hypertension patients are required to lifelong medication, which severely affects the living quality. Oral administration is preferred due to its convenience and safety. However, the oral antihypertensive peptides still face enormous difficulties, such as the inactivation by acid and enzyme in the GI (Richard, 2017), low effienciency in penetrateing the mucous layer of the intestinal (Lai et al., 2009), and unable to be efficiently absorbed by small intestinal epithelial cells, which result in the depressing therpeacy effect (Ensign et al., 2012). These defects seriously limit the application of antihypertensive peptides, thus it is essential to develop a new oral antihypertensive peptide system to solve the problem of poor stability (Chen et al., 2015) and achieve the goal of long-term effect of blood pressure reduction.

PLGA nanoparticles, as a safe drug-loading system, can effectively slow the drug release and protect drugs from gastrointestinal degradation (Lozoya-Agullo et al., 2017; Mustafa et al., 2017). Then coating the lipid membrane on the particles can further protect the drugs from GI and improve the contact between the nanoparticles and the small intestinal epithelial cells, which may achieve the goal of prolonging the circulation in the blood. The formed LNPs are potential to increase the stability, permeability and bioavailability of drugs (Zhang et al., 2010; Fang et al., 2014) in the GI.

The formed YF4-LNPs were prepared by a simple and controllable method (Yu et al., 2016). It was reported that EE and partice size both have enormous influence on the quality of nano-preparions (Johnstone et al., 2013). The utilization and absorption of drug can be improved by nano-preparions with higher EE and smaller particle size (Yu et al., 2016). Hence, factors which might influent the EE and size were optimized systemically, including the PLGA concentration, the volume of acetone in the organic phase, the pH of the aqueous medium, the volume of the inner aqueous medium, the external water phase, the PVA concentration, the PVA volume, the ultrasonic time, lipid material to nanomaterial ratio, and the ultrasound power. The an ever-growing PLGA viscosity in the organic phase caused droplets became larger and net shear stress became less, which would limit the free YF4 in inner aqueous diffuse into organic phase (Figure 1; Kiss et al., 2011; Turk et al., 2014). Therefore, the increasing PLGA concentration was indicated to lead to higher EE and larger particle size. The addition of acetone in the organic phase plays a great role in the nanoparticles forming (Anarjan et al., 2011). The addition of acetone in the organic phase could significantly affect the EE and particle size. The presence of acetone promoted organic phase to diffuse to inner phase (Luo et al., 2016), causing the carrier materials PLGA quickly precipitated and contained YF4 in the water phase, attributing to the EE increased. Whereas, too much acetone in the organic phase could also increase the opportunity of diffusion from inner to organic phase, which might result in the drug leakage (Yu et al., 2016). In addition, the interfacial energy at the oil/water interface was also reduced by the addition of acetone in the organic phase and thereby the stability of droplets was enhanced, which contributed to the smaller sizes of nanoparticles (Wang and Anderko, 2013). The opportunity of entrapment of the internal phase by the organic phase was enhanced as the W1/O volume ratio increased (Sang, 1999), which attribute to an excellent EE and a smaller particle size. However, when the W1/O volume ratio is too low or high, the W1/O emulsion structure was not complete, causing the poor EE and particle size. There is no doubt that the pH and ionizing status of peptide was highly related with the solubility of peptide (Ragab et al., 2004), which could affect the peptide loading state in the nanoparticles. When the pH was in isoelectric point (pI) of the YF4 peptide (Silva et al., 2013), in alkaline state, the state of charge distribution and the ionization state changed, the EE was significantly improved.

The effect of external water phase was also investigated. As an emulsifying agent, different external water phases make a great difference on the EE and particle size (Peng et al., 2007), which mainly related to viscosity and surface tension (Rayat and Feyzi, 2011). Although these surfactants can significantly reduce the surface tension, it is easy to lead to excessive viscosity of the aqueous phase, eventually leading to a larger particle size, the drug is not easy to be encapsulated, and the encapsulation efficiency is very low. Therefore, the optimal external aqueous media must possess these comprehensive properties to obtain higher EE and more appropriate particle size. According to above analysis, PVA was selected as the externalwater phase. Then we further evaluated the impacts of the concentration and volume of PVA. When the concentration of PVA was below 1%, the outer aqueous viscosity was too small to stable the droplet, the EE and the particle size were both unsatisfyed. However, the EE was declined as the increase of PVA concentration and volume (Song et al., 2008; Moralescruz et al., 2012; Sun et al., 2016), which might be attributed to the increase viscosity. In addition, the corresponding shear stress decreased as the increase viscosity, and led to the diffusion of YF4 from the inner aqueous phase into outside hampered, which might explain the lagrer particle size.

Ultrasonic duration might affect the drug leakage and the homogeneity of particle size in the preparation of LNPs. Moreover, peptides are generally denatured under high intensity ultrasonic shear systems (Gentile et al., 2012). Consequently, the proper ultrasonic duration was seemed as vital in the preparation of YF4-LNPs. The EE was increased firstly and suddenly decreased with longer ultrasonic time, which might be attributed by the broken coarse emulsion drops into nano-droplets (Fricker et al., 2010). However, too long ultrasonic time would lead to the leakage of YF4 from the internal phase to the external phase, causing a decrease in EE. Besides, prolonging W1/O/W2 ultrasonic time for a short time could significantly enhance the EE and decrease the particle size, caused by the increasing net sheer force, which is similar to the changes of ultrasonic power.

Ultimately, the ratio of PLGA/lipid materials was further optimized. As a result, a higher EE and a better particle size of YF4-LNPs were gained at 1:1.5 ratio of PLGA/lipid materials, which might result from the increased affinity between drug and the formulation (Luo et al., 2006).

To investigate the entrapment state of YF4 into lipid nanoparticles, DSC analysis were performed. The DSC showed that the YF4 was successfully entrapped in the YF4-LNPs. Additionally, the YF4-loaded nanoparticles were coated with lipid film according to TEM images, indicating the successful construction of lipid nanoparticles.

The release of YF4 from lipid nanoparticles was dramatically lower than the unincorporated YF4, which might be attributed to the surprising core-shell structure that could result in the tunable and sustained drug release profiles (Zhang et al., 2008; Fang et al., 2014). Although, our results are unsatisfactory, there is no obvious gastric protective and obviously sustained release in the intestinal tract, our study does protect the free drug from degradation in the stomach to a certain extent, providing more possibilities for its slow release in the intestinal tract and so as to prolonging the circulation in the blood and exerting its efficacy. Our result showed that only less than 30% of YF4 diffused into the dispersed medium after 12 h in all pH conditions, indicating a prolonged release of YF4-LNPs, which might lead to a potent and prolonged therapeutic efficacy of YF4-LNPs. For YF4-LNPs, the release percentages in media with different pH can provide some evidence that YF4-LNPs could provide sustainable release of YF4 in both stomach and intestine and protect YF4 from degradation.

It has been reported that ACEI peptides have amazing hypotensive activity *in vitro* (Escudero et al., 2012). We then firstly verified the BPL effect of YF4 compared with ACEI positive drug captopril shown in Figure 5 (Nurminen et al., 2000). However, the oral duration is short and the dosage of antihypertensive peptides is far higher than that in literature (Yu et al., 2016). Oral delivery of peptides has enormously continuous challenge due to its poor stability in the GI tract and low permeability through the intestinal epithelium membrane (Friedman and Amidon, 1991). The pharmacokinetics of YF4 in rats was studied and showed in the Supplementary Figure S1 and Supplementary Table 1. The results showed that the half-life of YF4 was only 2.91 h, just like that of conventional polypeptide and protein drugs. Thus, the new oral peptide delivery systems need to be developed. LNPs exhibit a wide range of erosion times, tunable biodegradation and mechanical properties, such as improving the connection between the nano-preparations and the intestinal epithelium cells, protecting the drugs from the GI, which might explain the antihypertensive effect in SHRs of YF4-LNPs. The YF4-LNPs exhibited a much better antihypertensive efficiency, especially YF4-LNPs showed a ten-time prolonged BPL effect. Currently, the mechanism of enhancement in therapeutic efficacy is still under investigation. The efficiency of YF4-LNPs being absorbed into systemic circulation requires further quantification. The association between elevated therapeutic efficacy and the increase in AUC or bioavailability needs further elucidation.

Over all, we systematically optimized the factors that could affect the features of LNPs, and finally constructed YF4-LNPs with a high entrapment efficiency and small particle size. Then, we evaluated its antihypertensive efficiency *in vivo*, and found that YF4-LNPs displayed stronger BPL effect and prolonged the effect up to 120 h. This may be explained by the controlled release of YF4 and the protection effect of the nanoparticles core and lipid membrane avoiding enzymatic degradation. This may provide some methodological clues and insights for the therapy of hypertension.

## Conclusion

In this study, we have developed uniform-sized PLGA lipid nanoparticles for oral delivery of peptides. The LNPs was successfully prepared by thin-film membrane hydration-ultrasonic dispersion method and systematically optimized. Then, the properties of YF4-LNPs were investigated, including the stability, the *in vitro* release profile. To our surprise, YF4-LNPs were considerable stable and showed a sustained release profile. Then the DSC curves and TEM illustrated that the successfully loading of YF4 and the coating of lipid bilayer. YF4-LNPs exhibited an enhanced antihypertensive function with a longer duration (up to 120 h in SHRs), which suggested the sustained release. In summary, all these data supported the belief that YF4-LNPs would be a promising platform for oral hypertension treatment. However, the preparation process of YF4-LNPs was relatively complex, which might be difficult to be scaled up. Hence, a simplified process is worthy of being further investigated to fabricateYF4-LNPs for the clinical translation study. Moreover, the pharmacokinetics of YF4-LNPs is also necessary to be investigated in the future.

## Author Contributions

JY conceived the project. XG, CF, and JY designed the experiments. SZ, JL, and YZ conducted most of the experiments. SZ and JL drafted the manuscript. LH performed some preliminary experiments. YL and JX participated in literature searching. JY, XG, and CF finished manuscript editing. All authors reviewed and approved the manuscript.

## Conflict of Interest Statement

The authors declare that the research was conducted in the absence of any commercial or financial relationships that could be construed as a potential conflict of interest.
