# Supplementary material for: Lipid Nanoparticles-Encapsulated YF4: A Potential Therapeutic Oral Peptide Delivery System for Hypertension Treatment
Source: Front Pharmacol. 2019 Feb 19;10:102. doi: 10.3389/fphar.2019.00102 (PMC6401629; doi:10.3389/fphar.2019.00102)
Supplement: Supplementary file 1 [file Table_1.docx]

# *Supplementary Materials*

# Lipid Nanoparticles-Encapsulated YF4: A Potential Therapeutic Oral Peptide Delivery System for Hypertension Treatment

Shengnan Zhao^1†^, Jinhua Li^1,2†^, Yang Zhou^1,2†^, Lingjing Huang^3^, Yanfei Li^3^, Juanjuan Xu^3^, Chunmei Fu^3^*, Xia Guo^2^*, Jian Yang^1,^*

^1^School of Applied Chemistry and Biological Technology, Shenzhen Polytechnic, Shenzhen, China

^2^Department of Pediatric Hematology/Oncology, Key Laboratory of Birth Defect and Related Disorders of Women and Children, West China Second University Hospital, Sichuan University, Chengdu, China

^3^Key Laboratory of Drug Targeting and Drug Delivery System (Ministry of Education), West China School of Pharmacy, Sichuan University, Chengdu, China

† These authors contributed equally to this work.

*****Correspondence: jiany@szpt.edu.cn (J.Y.); guoxkl@163.com (X.G.); fuchunmei@scu.edu.cn (C.F.)

## Graphical abstract


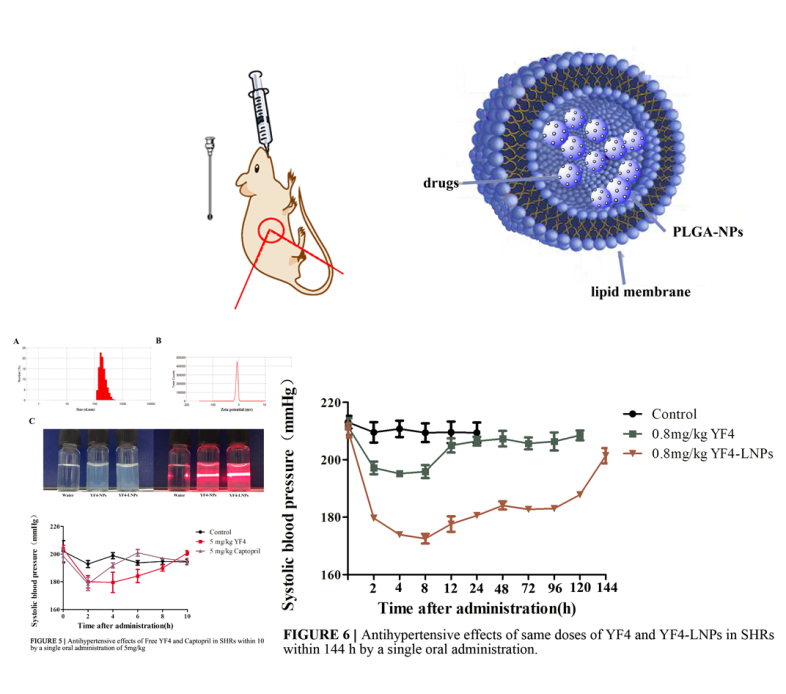


## Materials and methods

### Materials

YF4 (purity > 99%) was gained from Phtdpeptides Co., Ltd. (Zhengzhou, China).

### Animals

Six 8-week-old male SD rats with weight between 180 to 220 g were purchased from the Laboratory Animal Center of Sichuan University (Sichuan University, Chengdu, Sichuan, China). The animals were maintained under 12-hour dark and light cycles at 22℃ and free access to food and water. After a week of adaptation, these SD rats were admitted to experiments. Absorption of YF4 in SD rats following a single oral administration of YF4 was investigated. At the end of the experiment, the rats were sacrificed inside an isoflurane chamber to avoid unnecessary suffering. The experiments were approved and supervised by the State Key Laboratory of Biotherapy Animal Care and Use Committee (Sichuan University, Chengdu, Sichuan, China).

### In vivo pharmacodynamics study in SD rats

SD rats were used to investigate the pharmacodynamics of YF4. Rats were chosed randomly for oral administration of free YF4 at the dose of 1.2 mg/kg (n=6), respectively. Before the experiment, all rats were fasted for 12 h with free access to water. Each rat was administered an oral volume of 0.5 mL/100 g. Approximate 200 μL of blood samples were collected from the orbit into a heparin zed centrifuge tube before and at 0.083, 0.25, 0.5, 0.75, 1, 1.5, 2, 3, 4, 6, 8, 12 h post-dosing. All blood samples were centrifuged at 3500 rpm (4 ℃) for 10 min. The plasma samples were collected and stored at -20 ℃ until analyzed by Drug and Statistics 2.0 (DAS 2.0) (Mathematical Pharmacology Professional Committee of China, Shanghai, China).

### Stability of YF4-LNPs in different mediums

YF4-LNPs were diluted with different pH solutions at 37°C for 12 h to measure its stability in different solutions. The changes of particle size and EE of YF4-LNPs were examined by the methods described above. Analyses were performed in triplicate and the values were expressed as mean ± S.D.

### Statistical analysis

The obtained data were analyzed using the Graph Pad Prism 5. Data were analyzed by one-way analysis of variance. p<0.05 was considered a statistically difference, and p < 0.01 was considered a statistically significant difference.


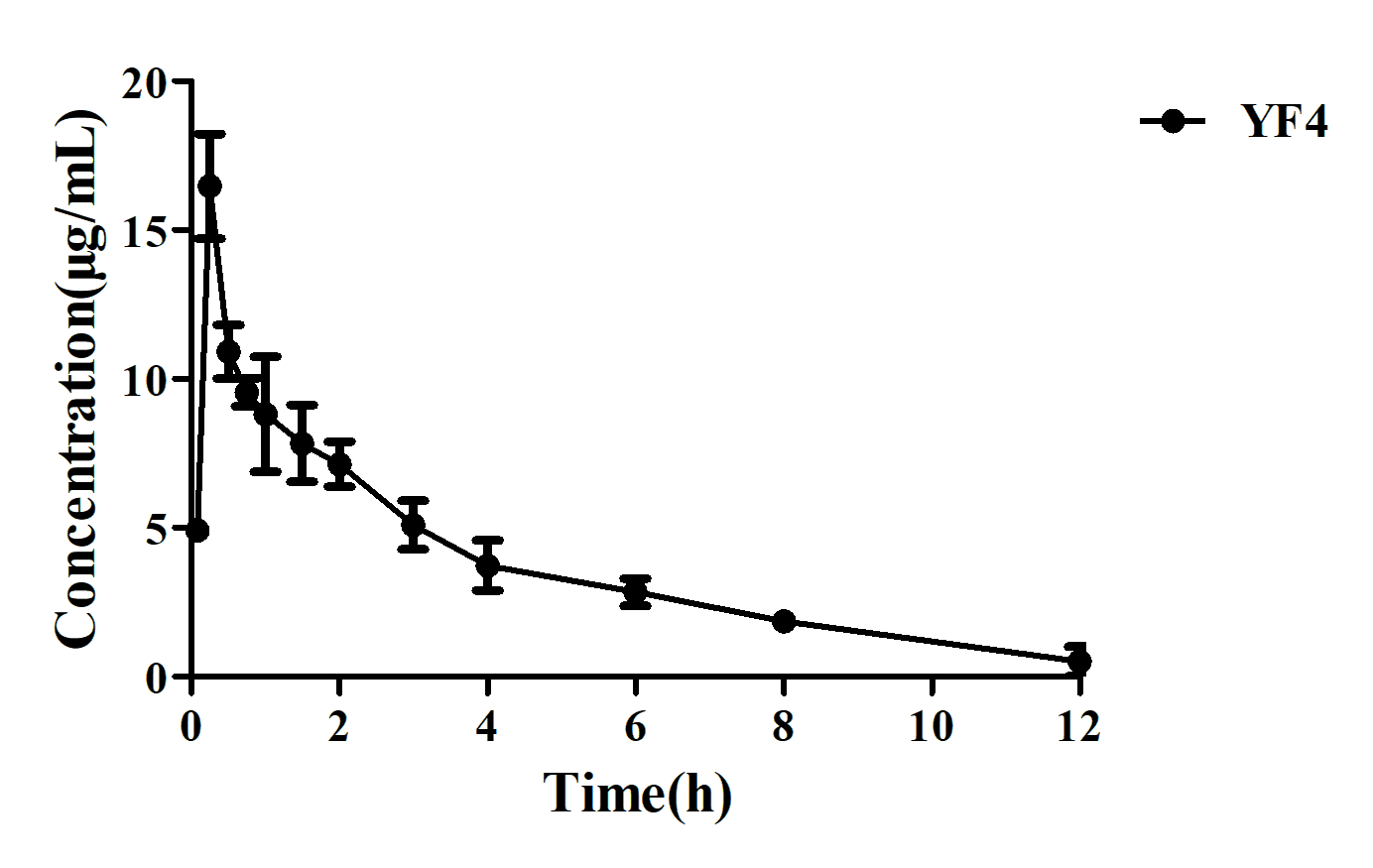


**Figure S1** Plasma concentration-time curves from SD rats treated with Free YF4, after a single gavage administration (n=6).

**Table 1.** Pharmacokinetic parameters after a single gavage administration in SD rats. Data are expressed as mean ± SD (n = 6).

|  | Unit | YF4 |
| --- | --- | --- |
| AUC_(0-72h)_ | mg/L*h | 38.85±10.86 |
| AUC_(0-∞)_ | mg/L*h | 45.39±9.42 |
| T_1/2z_ | h | 2.91±0.05 |
| C_max_ | mg/L | 15.47±3.05 |
| CLz/F | L/h/kg | 0.02±0.00 |
| MRT_(0-t)_ | h | 3.17±0.68 |


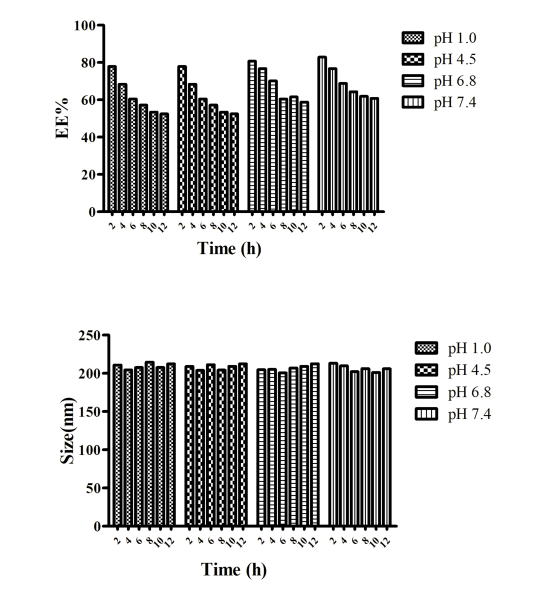


**Figure S2** Stability of YF4-LNPs in different mediums (n=3).
